# Metabolic Effects of the Cancer Metastasis Modulator MEMO1

**DOI:** 10.3390/metabo15040277

**Published:** 2025-04-17

**Authors:** Marziyeh Ghanbarian, Natalia Dolgova, Frederick S. Vizeacoumar, Franco J. Vizeacoumar, Deborah Michel, Anas El-Aneed, Oleg Y. Dmitriev

**Affiliations:** 1 Department of Biochemistry, Microbiology and Immunology, University of Saskatchewan, Saskatoon, SK S7N 5E5, Canada; marzi.gh@usask.ca (M.G.);; 2 Department of Pathology and Laboratory Medicine, University of Saskatchewan, Saskatoon, SK S7N 5E5, Canada; frederick.vizeacoumar@usask.ca; 3 Cancer Research Department, Saskatchewan Cancer Agency, Saskatoon, SK S7N 5E5, Canada; 4 Division of Oncology, University of Saskatchewan, Saskatoon, SK S7N 5E5, Canada; 5 College of Pharmacy and Nutrition, University of Saskatchewan, Saskatoon, SK S7N 5E5, Canada

**Keywords:** MEMO1, tricarboxylic acid cycle, metal binding protein, cancer metastasis, LC-MS/MS, iron regulation, breast cancer, energy metabolism

## Abstract

**Background/Objectives:** Cancer cells often display altered energy metabolism. In particular, expression levels and activity of the tricarboxylic acid cycle (TCA cycle) enzymes may change in cancer, and dysregulation of the TCA cycle is a frequent hallmark of cancer cell metabolism. MEMO1, a modulator of cancer metastasis, has been shown to bind iron and regulate iron homeostasis in the cells. *MEMO1* knockout changed mitochondrial morphology and iron content in breast cancer cells. Our previous genome-wide analysis of *MEMO1* genetic interactions across multiple cancer cell lines revealed that gene sets involved in mitochondrial respiration and the TCA cycle are enriched among the gain-of-function interaction partners of *MEMO1*. Based on these findings, we measured the TCA cycle metabolite levels in breast cancer cells with varying levels of *MEMO1* expression. **Methods:** ShRNA knockdown assay was performed to test essentiality of key TCA cycle enzymes. TCA metabolites were quantified using liquid chromatography-tandem mass spectrometry (LC-MS/MS) in *MDA-MB-231* (high *MEMO1*), *M67-2* (*MEMO1* knockdown), and *M67-9* (MEMO1 knockout) cells under iron-depleted, basal iron, and iron-supplemented conditions. **Results:** *ACO2* and *OGDH* knockdowns inhibit cell proliferation, indicating an essential role of the TCA cycle in *MDA-MB-231* metabolism. α-Ketoglutarate and citrate levels exhibited an inverse relationship with *MEMO1* expression, increasing significantly in *MEMO1* knockout cells regardless of iron availability. In contrast, fumarate, malate, and glutamate levels were elevated in *MEMO1* knockout cells specifically under low iron conditions, suggesting an iron-dependent effect. **Conclusions:** Overall, our results indicate that MEMO1 plays a role in regulating the TCA in cancer cells in an iron-dependent manner.

## 1. Introduction

MEMO1, a highly conserved cytosolic protein, was initially identified for its involvement in cell motility and various cellular processes, including vitamin D metabolism and central nervous system development [1,2,3,4]. The significance of *MEMO1* in cancer is underscored by its overexpression across multiple malignancies, such as breast, colon, lung, and uterine cancers, as evidenced by analyses of the Cancer Genome Atlas database (https://www.cancer.gov/tcga, accessed on 18 January 2024). This overexpression is particularly pronounced in aggressive breast cancer subtypes, including triple-negative and HER2-positive (HER2+) cancers, which are associated with poor patient outcomes and limited treatment options [5]. The contribution of MEMO1 to carcinogenesis is mediated through interactions with the insulin receptor substrate protein 1 pathway and extranuclear estrogen receptor signaling in breast cancer [6,7]. Importantly, MEMO1 appears to promote cancer metastasis, particularly breast cancer by regulating microtubule dynamics and enhancing lamellipodia formation, thereby enabling cancer cells to migrate into surrounding tissues [3,8].

In addition to its role in cell motility, MEMO1 has recently emerged as a significant regulator of iron homeostasis [5]. A genome-wide in silico analysis of publicly available gene essentiality data from 1028 cancer cell lines has identified numerous proteins involved in iron metabolism that display statistically significant genetic interactions (GIs) with *MEMO1*. Notably, this includes proteins such as transferrin receptor 2 (TFR2), mitoferrin-2 (SLC25A28), and the iron response regulator IRP1 (ACO1), which are involved in iron uptake, transport, and regulation within mitochondria [5]. Iron dysregulation is central to cancer progression, driving redox imbalance and fueling the high metabolic demands of proliferating cells [9]. Thus, MEMO1 can serve as a critical link between iron metabolism and metastasis, particularly in breast cancer.

Measurements of iron concentration in breast cancer cells with varying *MEMO1* expression revealed distinct mitochondrial iron levels. *MEMO1*-overexpressing cells exhibited increased iron content in the mitochondrial fraction compared to the *MEMO1*-knockout cells [5]. These observations imply that MEMO1 is involved in iron maintenance to sustain the proliferation of high-*MEMO1* cells with heightened iron demands. Additionally, *MEMO1*-deficient cells exhibited abnormal mitochondrial distribution and morphology, characterized by perinuclear clustering under iron depletion conditions [5]. Taken together, these findings suggest that MEMO1 has a specific function in regulating iron levels within the mitochondria, beyond its broader role in overall iron homeostasis in cancer cells.

To better understand the role of MEMO1 in mitochondrial function we have conducted a genome-wide analysis of *MEMO1* genetic interactions using gene essentiality data contained in several databases of single-gene knockouts and knockdowns in cancer cells [5]. Remarkably, gene set enrichment analysis (GSEA) of the gain-of-function genetic interactions (GOF-GIs), i.e., interactions displayed in high-*MEMO1* cells, identified several gene sets related to the mitochondrial energy metabolism, including Respiratory Electron Transport Chain (false discovery rate (FDR) of 1.54 × 10^−2^), TCA Cycle and Respiratory Electron Transport (FDR 5.99 × 10^−3^), and Oxidative Phosphorylation (FDR 4.32 × 10^−2^). In total, we identified approximately one hundred GOF-GIs related to the TCA cycle, electron transport chain, and oxidative phosphorylation. The multiple GOF interactions between MEMO1 and proteins involved in these processes indicate that high-*MEMO1* cells are particularly sensitive to disruptions of energy metabolism. This finding prompted us to investigate the link between MEMO1 and the TCA.

TCA cycle, also known as the Krebs or citric acid cycle, is a central metabolic hub for the oxidative metabolism of nutrients, including glucose, fatty acids, and amino acids, to support cellular bioenergetics [10,11]. It functions in both catabolic and anabolic capacities and is therefore considered amphibolic [12,13]. Each turn of this cycle involves the transfer of high-energy electrons from reducing metabolites to NAD^+^ and FAD as key electron carriers. These carriers then transfer the electrons to the complexes of the electron transport chain, and then finally to oxygen, coupled with the generation of the proton motive force required to produce ATP through oxidative phosphorylation (OXPHOS) in mitochondria [14].

In cancer cells, the TCA cycle often undergoes significant change during general metabolic reprogramming, which allows cancer cells to meet their metabolic needs at every stage of tumor growth and metastasis progression [15,16]. This reprogramming provides metabolic flexibility to rewire intracellular metabolic pathways to serve the altered bioenergetic and biosynthetic demands of rapidly proliferating cancer cells under diverse nutrient conditions and microenvironments [17,18]. One of the most well-known examples of metabolic reprogramming in cancer cells is the Warburg effect, in which cancer cells preferentially utilize glycolysis over mitochondrial respiration for ATP generation under aerobic conditions [19]. However, it is now recognized that some cancer cells do utilize OXPHOS as a major means of ATP generation [20]. Despite its lower ATP yield compared to oxidative phosphorylation, the preference for glycolytic metabolism in cancer cells underscores a critical shift in cellular priorities [21]. Cancer cells prioritize the synthesis of essential biomolecules and maintain redox balance to maximize ATP production. Heightened glycolytic activity ensures rapid glucose uptake and channeling of glycolytic intermediates into anabolic pathways [18,22]. Even when glycolysis is enhanced, the TCA cycle remains indispensable, serving as a nexus for the metabolic exchange and conversion of glucose, lipids, and certain amino acids. Altered levels of the essential TCA cycle intermediates such as citrate, α-ketoglutarate, succinate, and fumarate have been implicated in tumorigenesis and tumor progression, modulating the tumor metabolism, signal transduction and immune environment [22].

To sustain the TCA cycle function, cancer cells employ diverse strategies, demonstrating key aspects of their metabolic flexibility, such as glutaminolysis for anaplerosis and reductive carboxylation for lipid synthesis [23,24]. When the TCA intermediates are drawn off for biosynthetic needs, or when glucose-derived pyruvate is limited, cells use glutaminolysis, a process where glutamine deamination is followed by glutamate oxidation to supply α-ketoglutarate (α-KG) to fuel the TCA cycle [25,26,27]. Under hypoxic conditions or in tumors with mitochondrial defects, cells also reduce their reliance on the conventional oxidative TCA cycle and use glutamine to generate α-ketoglutarate, allowing the TCA cycle to function via alternative pathways. α-KG can then be reductively carboxylated to citrate, which is subsequently used for lipid synthesis [28,29]. ATP citrate lyase (ACL)-mediated citrate cleavage also serves as another metabolic adaptation to bypass the truncated TCA cycle while maintaining essential biosynthetic pathways by providing an alternative route for oxaloacetate (OAA) production [24].

The enhanced rates of glycolysis and TCA cycle activity in cancer cells can be partly attributed to oncogenic mutations and the deregulation of signaling pathways. Specifically, key oncogenes like *c-Myc* and *KRAS* transactivate lactate dehydrogenase A (*LDHA*) and promote the expression of glutamate oxaloacetate transaminase 1 (*GOT1*), respectively [30,31]. PI3K/AKT mediated growth factor signaling activates glucose metabolism and induces aerobic glycolysis through direct phosphorylation or allosteric regulation of glycolytic enzymes [32,33]. Phosphorylation of pyruvate dehydrogenase by AMP-activated protein kinase (AMPK) significantly stimulates the TCA activity and promotes cancer metastasis [34]. Similarly, the OGT–c-Myc–PDK2 axis has been implicated in the TCA cycle rewiring, a key adaptation that fuels tumor growth in colorectal cancer [35]. Additionally, mutations affecting the integrity of the TCA cycle, especially those involving isocitrate dehydrogenase (*IDH*), succinate dehydrogenase (*SDH*), and fumarate hydratase (*FH*), lead to dysregulation in the production of the TCA cycle metabolites, known as oncometabolite accumulation [36,37]. This process has been linked to cancer onset by inhibiting histone and DNA demethylases, leading to altered gene expression patterns and cancer cell stemness [38,39].

These findings led us to a hypothesis that variations in *MEMO1* expression levels can cause alterations in cancer metabolism, especially in the TCA cycle, and that these changes are further modulated by iron availability. To test this hypothesis, we employed a targeted metabolomics approach to quantify key TCA cycle metabolites, including citrate, fumarate, malate, α-KG, glutamate, pyruvate, and succinate using liquid chromatography–tandem mass spectrometry (LC-MS/MS) to obtain a comprehensive snapshot of the TCA cycle activity. Metabolite levels were measured in triple-negative breast cancer cell lines with high, low and no *MEMO1* expression. Next, to test whether iron availability modulates MEMO1-dependent TCA cycle changes, we compared the metabolic profiles of cells with different *MEMO1* levels under basal iron concentration in the growth medium, iron depletion and subtoxic iron excess.

## 2. Materials and Methods

### 2.1. Identification of MEMO1 Genetic Interactions Related to the TCA Cycle

The genome wide list of *MEMO1* genetic interactions was generated by screening several public databases of single gene knockouts and knockdowns in multiple cancer cell lines as described in detail previously [5]. To identify genes related to the TCA cycle, following the gene set enrichment analysis [5], the full list of *MEMO1* genetic interactions was searched for the genes contained in the KEGG [40] Citrate Cycle TCA Cycle gene set using a gawk script.

### 2.2. Cell Lines, shRNA Assay and Western Blot

*MDA-MB-231* (breast cancer) cell line was obtained from ATCC and cultured in Dulbecco’s Modified Eagle Medium (DMEM, HyClone) supplemented with 10% fetal bovine serum (FBS, Gibco). *MEMO1*-knockdowns (*MEMO1*-KDs) and *MEMO1*-knockouts (*MEMO1*-KOs) were generated using CRISPR-Cas9 technology, as described previously [5]. ShRNA was delivered to *MDA-MB-231*, *M67-2*, and *M67-9* cells by lentiviral transfection, and the virus was removed after a 24-h incubation. RFP-shRNA was used as a control. Following the incubation with puromycin for the next 48 h, the cells were trypsinized and plated at 2000 cells per well in 96-well plates in eight replicates. Imaging was performed every 8 h using the Incucyte^®^S3 Live-Cell Analysis Instrument (Sartorius Bioanalytical Instruments, Inc. Ann Arbor, MI, USA) for 120–140 h. Proliferation rates were calculated by fitting cell confluency data using the logistic growth equation in GraphPad Prism v.9, as described previously [5]. To confirm shRNA-mediated knockdown of *OGDH* and *ACO2*, Western blot analysis was performed on the remaining cells harvested from 100 mm plates after 48 h.

### 2.3. Sample Preparation for LC-MS/MS

Cells were cultured in 100 mm plates (three biological replicates) and were treated either with no additions (further referred to as basal Fe^2+^ level), with 1 µM deferoxamine (DFX) as an iron chelator (low Fe^2+^), or 10 µM ferric citrate as iron supplement (high Fe^2+^) for 24 h. These concentrations were determined as the maximum non-toxic doses using a resazurin fluorescence cell viability assay [5]. Next, cells were rinsed with PBS, pH 7.2, trypsinized, and a volume equivalent to 4 × 10^6^ cells (*n* = 3) was transferred into a microtube. Cells were pelleted by centrifugation at 200× *g* for 5 min, the supernatant was removed, and the pellet was resuspended in 1 mL cold PBS, pH 7.2, followed by another centrifugation at 300× *g* for 5 min to remove residual media. The pellet was then resuspended in 400 µL of 80% (*v*/*v*) cold methanol. The samples were subjected to probe sonication (CPX-130 Ultrasonic Processor, Cole-Parmer Instrument Co., Vernon Hills, IL, USA) and centrifuged at 14,000× *g* for 10 min. The supernatant was transferred to a fresh microtube and aliquoted into volumes equivalent to 1 × 10^6^ cell count. The aliquots were then dried using Labconco SpeedVac concentrator (Labconco Corporation, Kansas City, MO, USA).

### 2.4. Metabolomic Analysis

A targeted high-performance liquid chromatography–tandem mass spectrometry (HPLC-MS/MS) method utilizing dimethylaminophenacyl (DmPA) derivatization with ^12^C/^13^C isotope labeling was employed for the quantification of citrate, fumarate, malate, α-KG and glutamate [41,42], while stable isotope-labeled precursors were directly incorporated to quantify pyruvate (^13^C_3_-pyruvic acid, Omicron Biochemical. Inc, USA) and succinate (succinic-d_4_ acid, MedChem Express, Monmouth Junction, NJ, USA) [43]. Following DmPA derivatization, the ^13^C_2_-DmPA-labeled metabolites were added as internal standards, and the samples were analyzed using a Kinetex C18 reverse-phase 5 μm × 100 mm, 2.1 mm ID column (Phenomenex, Torrance, CA, USA) on an Agilent 1260 Series HPLC system coupled with an AB SCIEX 4000 QTRAP^®^ mass spectrometer (AB Sciex, Concord, ON, Canada), equipped with a quadrupole-linear ion trap and electrospray ionization (ESI) source. A previously described binary mobile phase composition was used [41], with a 12-min gradient and a flow rate of 250 µL/min. Quantification achieved in positive mode with multiple reaction monitoring (MRM) with an ion spray voltage (ISV) of 5500 V. The precursor ions (*m*/*z*) for these derivatized metabolites were previously reported [42].

For the analysis of succinate and pyruvate, a hydrophilic interaction liquid chromatography–tandem mass spectrometry (HILIC-MS/MS) method was applied. Isotope labeled metabolites were added as internal standards, and the chromatographic separation was performed using a SeQuant^®^ ZIC^®^-HILIC™ 3.5 μm × 150 mm, 2.1 mmID column (MilliporeSigma, Burlington, MA, USA) on an Agilent 1290 Series UHPLC system coupled with AB Sciex 6500 API QTRAP^®^ (AB Sciex, Concord, ON, Canada) mass spectrometer, equipped with a Q-LIT and ESI, operating in MRM mode with negative ionization with an ISV of −4500 V. The gradient mobile phase consisted of Solvent A and Solvent B, as previously described [44], with a gradient time of 7.2 min and a flow rate of 500 μL/min. Full details, including the precursor ions (*m*/*z*) and applied collision energies, can be found in the Metabolights deposition (accession number: MTBLS12348).

Data processing was conducted using Analyst^®^ software, version 1.7 (AB Sciex, ON, Canada). Mass spectral data were acquired from three independent biological replicates, each with three technical replicates. One-way ANOVA was conducted using GraphPad Prism version 10.4.0, and statistically significant differences (*p* < 0.05) between the parental cell line, *MEMO1* knockdown, and *MEMO1* knockout under different iron availability conditions were reported.

All solvents used in this experiment were Optima^®^ LC/MS grade and purchased from Fisher Scientific (Ottawa, ON, CA). Metabolites were obtained at analytical grade from commercial sources. ^12^C_2_-dimethylaminophenacyl bromide (^12^C_2_-DmPA-Br) was sourced from Combi-Blocks Inc. (San Diego, CA, USA), while ^13^C_2_-dimethylaminophenacyl bromide (^13^C_2_-DmPA-Br) was synthesized in-house as described previously [41].

## 3. Results

### 3.1. MEMO1 GOF-GIs Highlight a Link to Mitochondrial Energy Metabolism and Redox Homeostasis

**Figure 1:**
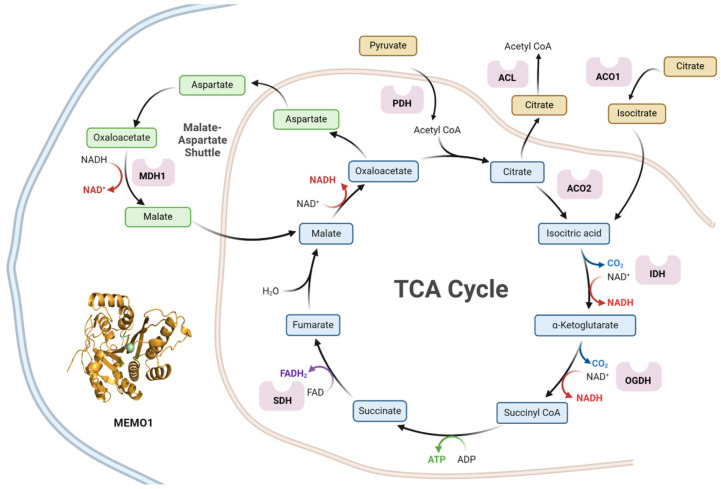
Tricarboxylic acid cycle (TCA cycle, Krebs cycle) with enzymes exhibiting gain-of-function genetic interactions with *MEMO1* in our analysis, including ATP-citrate lyase (ACL), aconitase 1 (ACO1), aconitase 2 (ACO2), isocitrate dehydrogenase (IDH), oxoglutarate dehydrogenase (OGDH), succinate dehydrogenase (SDH), malate dehydrogenase 1 (MDH1), and pyruvate dehydrogenase (PDH). The double light blue and brown lines represent the mitochondrial and cytoplasmic boundaries, respectively. The figure was created with BioRender (web-based application).

We have previously found that the TCA cycle and Respiratory Electron Transport gene set is enriched among *MEMO1* gain-of-function interactions. To further characterize the TCA cycle component of the *MEMO1* GOF-GI network (Figure 1), we screened the complete genome wide list of *MEMO1* GOF-GIs and identified eighteen genes specifically linked to the TCA cycle activity, demonstrating significant *p*-values (*p* < 0.05) and high differential essentiality (high/low distance) scores (Table 1).

### 3.2. MDA-MB-231 Cells Metabolism Relies on the TCA Cycle: OGDH and ACO2 Knockdowns Inhbit Cell Proliferation

**Figure 2:**
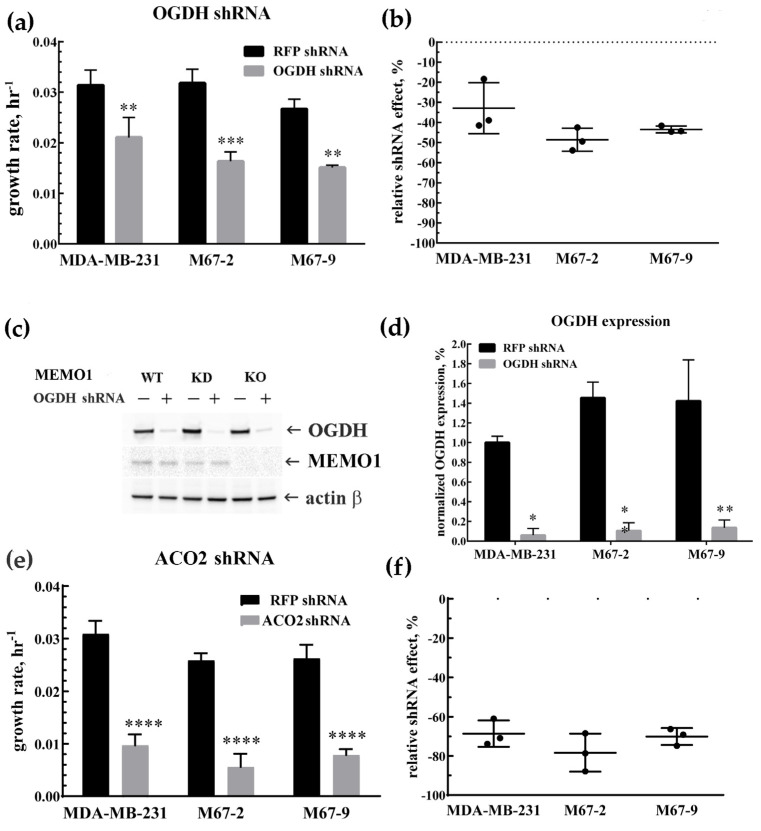
Knockdowns of *OGDH* and *ACO2* inhibit cell proliferation at all *MEMO1* expression levels. Effects of *OGDH* (**a**,**b**) and *ACO2* (**e**,**f**) shRNA knockdowns on the cell proliferation rates at different *MEMO1* expression levels (*MDA-MB-231*—parental cell line, *M67-2*—*MEMO1* knockdown, *M67-9*—*MEMO1* knockout). OGDH protein levels were detected by Western blot (**c**,**d**). ACO2 levels were not tested due to nearly complete proliferation inhibition. The relative shRNA effect on cell proliferation (**b**,**f**) is expressed as the difference between the proliferation rates with the shRNA targeting the gene of interest and the control shRNA (against the red fluorescent protein) divided by the proliferation rate with the control shRNA. Statistically significant differences vs. the parental cell line are marked (* for *p* < 0.05, ** for *p* < 0.01, *** for *p* ˂ 0.001 and **** for *p* ˂ 0.0001).

Prior computational analysis of *MEMO1* GOF-GIs identified *OGDH* and *ACO2* as interacting genes associated with mitochondrial function and involved in the TCA cycle. To validate these interactions experimentally and to test the role of these key TCA enzymes in the cell metabolism at various *MEMO1* expression levels, we measured proliferation rates in *MEMO1*-expressing (*MDA-MB-231*), *MEMO1*-knockdown (*M67-2*), and *MEMO1*-knockout (*M67-9*) cells following shRNA-mediated knockdown of *OGDH* and *ACO2* (Figure 2). The knockdown assays revealed a significant decrease in cell proliferation rates across all cell lines, regardless of *MEMO1* expression levels. The *OGDH* knockdown reduced cell proliferation rate by 30–50%. Consistent with GOF-GI, the effect appeared to be stronger in *MEMO1*-KD and *MEMO1*-KO cells, but the difference did not reach statistical significance threshold in our experiments. By comparison, *ACO2* knockdown inhibited the cell proliferation almost completely. Taken together, these results indicate that *MDA-MB-231* cells critically rely on the TCA for their metabolic needs, as demonstrated by the essential role of ACO2. The less severe effect of the *OGDH* knockdown suggests a supplemental pathway for succinyl-CoA generation, perhaps through amino acid catabolism.

### 3.3. MEMO1-Mediated Modulation of Mitochondrial Metabolism via Iron Homeostasis

To experimentally investigate the link between MEMO1 and the TCA activity suggested by our bioinformatic analyses, we employed a targeted metabolomics approach to measure key TCA metabolites in the *MDA-MB-231* triple-negative breast cancer cell line, which exhibits high *MEMO1* expression. Specifically, citrate, fumarate, malate, α-KG, glutamate, pyruvate, and succinate levels were quantified and compared across isogenic *MDA-MB-231*-derived *MEMO1* knockdown (*M67-2*) and *MEMO1* knockout (*M67-9*) cell lines, generated previously [5].

Metabolite quantification was achieved through HPLC-MS/MS with isotopically labeled analytes derivatized with DmPA for five of the metabolites, while the remaining two were analyzed by HILIC-MS/MS without derivatization. To determine whether iron availability modulates the interplay between MEMO1 and the TCA cycle activity, cells were treated with either 1 µM deferoxamine (DFX), an extracellular iron chelator, to induce iron depletion, or with 10 µM ferric citrate for iron supplementation, the highest non-toxic concentrations, based on prior cytotoxicity assays [5]. These conditions, along with a basal iron condition, were compared to assess the impact of iron on the metabolite profiles.

#### 3.3.1. Citrate Concentrations Exhibit a Consistent Trend at the Basal Iron Level

**Figure 3:**
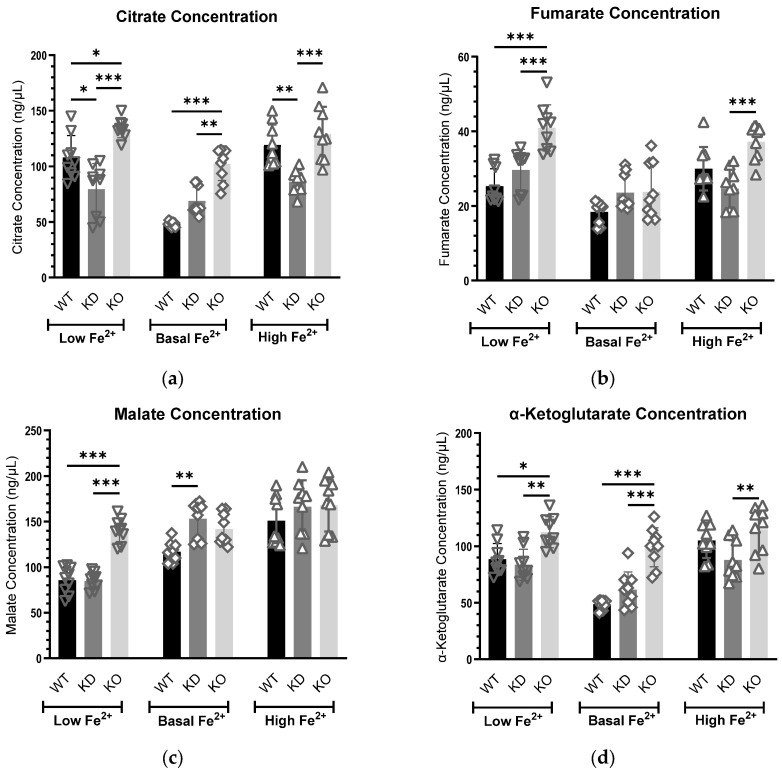

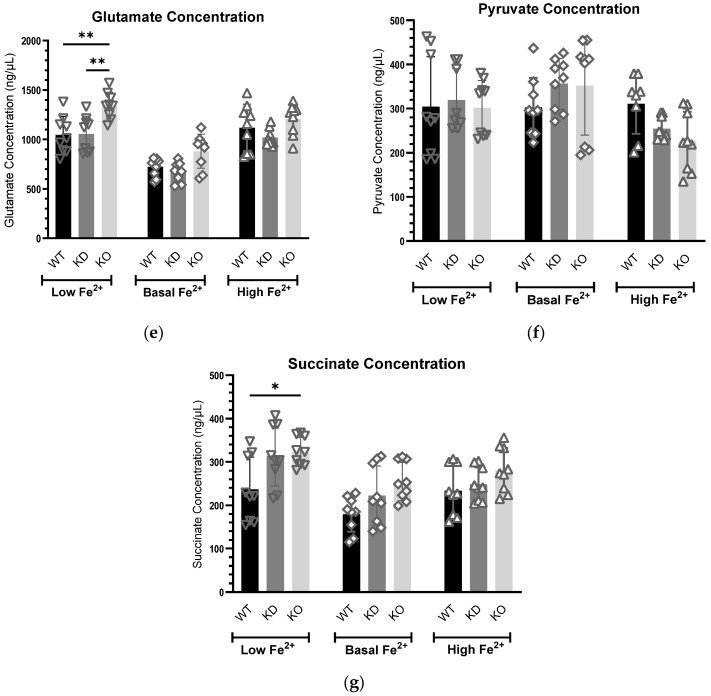
TCA metabolite levels in breast cancer cells at various iron and *MEMO1* levels. Comparison of citrate (**a**), fumarate (**b**), malate (**c**), α-ketoglutarate (**d**), glutamate (**e**), pyruvate (**f**), and succinate (**g**) at different *MEMO1* expression levels (WT—parental cell line, KD-*MEMO1* knockdown, KO-*MEMO1* knockout) under iron-deficient (low Fe^2+^), normal (basal Fe^2+^), and iron overload (high Fe^2+^) conditions. To induce iron depletion and supplementation, all three cell lines were treated with 1 μM deferoxamine and 10 μM ferric citrate, respectively, for 24 h. Downward-pointing triangles (▽) represent low Fe^2+^, diamonds represent basal Fe^2+^ (◇), and upward-pointing triangles represent high Fe^2+^ (△). Data were collected from three independent biological replicates, each with three technical replicates. Statistically significant differences compared to the parental cell line (WT) are indicated (* for *p* < 0.05, ** for *p* < 0.01, and *** for *p* < 0.001).

Citrate concentrations exhibited a consistent inverse relationship with *MEMO1* expression level, following the pattern: high-*MEMO1* cells < *MEMO1*-KD cells < *MEMO1*-KO cells under basal iron conditions (Figure 3a). This increase was statistically significant between either parental high-*MEMO1* or *MEMO1*-KD cells and *MEMO1*-KO cells. At both high iron and iron depletion conditions, citrate concentrations were significantly lower in *MEMO1*-KD cells than in either high *MEMO1* or *MEMO1*-KO cells. These differences indicate a complex relationship between MEMO1 and iron in regulating citrate concentration in the cell.

#### 3.3.2. Fumarate Levels Are Elevated in *MEMO1*-Deficient Cells Under Altered Iron Conditions

Under perturbed iron conditions, fumarate levels exhibited a progressive increase, reaching significantly higher concentrations in *MEMO1*-null cells compared to both high-*MEMO1* and *MEMO1*-KD cells, particularly under low iron conditions (Figure 3b). This suggests that iron deficiency exacerbates fumarate accumulation in the absence of MEMO1. In addition, a statistically significant elevation in fumarate concentration in *MEMO1*-KO cells under iron supplementation implies that MEMO1 may influence fumarate metabolism under both iron-deficient and iron-replete conditions. In contrast, fumarate levels remained comparable across all cell types under basal conditions, suggesting that MEMO1’s regulatory role in fumarate metabolism becomes more pronounced under iron stress.

#### 3.3.3. MEMO1 Regulates Malate Levels Primarily Under Iron Limitation

Under iron-deficient conditions, malate concentrations were significantly elevated in *MEMO1*-KO cells compared to both *MEMO1* knockdown and parental high-*MEMO1* cells (Figure 3c). This may indicate that MEMO1 plays a critical role in regulating malate metabolism, particularly when iron availability is limited. In contrast, at elevated iron levels, no significant changes in malate levels were detected across these cell types. At basal iron levels, a small but statistically significant difference was detected between high-*MEMO1* and *MEMO1*-KD cells.

#### 3.3.4. *MEMO1* Loss Consistently Elevates α-Ketoglutarate Levels, Indicating Disrupted α-KG Metabolism

α-KG concentration was generally elevated in *MEMO1* knockout (KO) cells across all conditions. This may suggest that MEMO1 loss disrupts α-KG metabolism or that MEMO1 normally functions to regulate or constrain α-KG levels within the cell, regardless of iron availability (Figure 3d). Under basal conditions, this elevation followed a trend inversely related to *MEMO1* expression levels, similar to citrate. Specifically, *MEMO1*-KO cells exhibited a statistically significant increase in α-KG relative to both *MEMO1* knockdown (KD) and *MEMO1*-overexpressing cells, highlighting a potential inverse dose-dependent relationship between *MEMO1* expression and α-KG levels under normal growth conditions. This trend persisted under low iron conditions with a smaller difference between *MEMO1* expression levels.

#### 3.3.5. Iron Deficiency Affects Glutamate Metabolism in *MEMO1* Knockout Cells

Under iron-deficient conditions, a significant increase in glutamic acid levels was observed in *MEMO1*-KO cells compared to both parental high-*MEMO1* expressing cells and *MEMO1* knockdown cells (Figure 3e). No marked changes in glutamic acid levels were detected across these cell types under other iron conditions. This suggests that MEMO1 loss specifically disrupts glutamate metabolism under iron restriction, indicating a potential interplay between iron availability and MEMO1 function in the regulation of glutamate metabolism.

#### 3.3.6. Pyruvate and Succinate Concentrations Remain Stable Despite Variations in *MEMO1* Levels and Iron Availability

Pyruvate and succinate concentrations remained stable across different *MEMO1* expression levels and varying iron availability conditions, indicating that neither *MEMO1* expression nor iron status significantly affects the concentrations of these metabolites. However, under low iron conditions, a small but statistically significant difference was observed in succinate concentration between *MEMO1*-KO and parental high *MEMO1* cells. This subtle difference may reflect anomalous data variance in this dataset rather than a true biological effect (Figure 3f,g).

## 4. Discussion

MEMO1, an evolutionarily conserved protein implicated in cancer, binds iron and regulates iron metabolism, thereby influencing tumor cell proliferation, metastasis, and sensitivity to ferroptosis, particularly in breast cancer [5]. While MEMO1 role in iron homeostasis is well established, the precise mechanisms by which it regulates downstream iron-related metabolic pathways remain unclear. As we started exploring these mechanisms, our recent GSEA of the computationally derived *MEMO1* GOF-GIs revealed a strong link between MEMO1, mitochondrial energy metabolism, and redox balance in the cell [5]. Given the enrichment of the TCA-cycle related genes in these datasets, it is likely that MEMO1 role in iron homeostasis is linked to metabolic adaptations in the TCA cycle in cancer cells.

This connection is particularly relevant as cancer cells demonstrate remarkable flexibility in the TCA cycle activity to meet their metabolic demands for survival and proliferation. Cancer cells dynamically adjust substrate preferences based on nutrient availability, utilizing glucose, glutamine, and lactate as primary fuels [50,51,52]. They also regulate cataplerosis (channeling of the TCA cycle intermediates for biosynthetic processes) and anaplerosis (replenishment of the TCA cycle intermediates) to maintain the TCA cycle function [12]. Under hypoxic conditions, when acetyl-CoA production is limited, cancer cells employ reductive carboxylation of α-ketoglutarate to citrate as an alternative pathway for lipid synthesis [24,28]. Given that MEMO1 regulates cellular iron levels, particularly within mitochondria, and shows genetic interactions with many iron-related proteins, including those involved in the TCA cycle, it is plausible that MEMO1 modulates the TCA cycle activity in an iron-dependent manner.

To investigate the link between MEMO1 and the TCA, we assessed concentration changes of the key TCA metabolites relative to *MEMO1* expression levels and iron status. Metabolite quantification was performed using isotope-labeled liquid chromatography-tandem mass spectrometry (LC-MS/MS) in a panel of isogenic breast cancer cell lines, derived from *MDA-MB-231*, a high-*MEMO1* cell line originating from a TNBC tumor, alongside *MEMO1*-knockdown (*M67-2*) and *MEMO1*-knockout (*M67-9*). Cells were cultured under three iron conditions: 1 µM deferoxamine (low Fe^2+^), 10 µM ferric citrate (high Fe^2+^), and untreated control conditions (basal Fe^2+^).

The metabolites selected for analysis play well-established roles in cancer, particularly in tumor metabolism and signal transduction. Fumarate and succinate are oncometabolites, and α-ketoglutarate is a tumor suppressor, all of which participate in the regulation of α-KG-dependent dioxygenase activity, impacting both epigenetic and metabolic processes in cancer [53]. Citrate metabolism is frequently altered in tumors and has been associated with both tumorigenesis and inhibition of cancer cell proliferation [54,55]. Malate plays a key role in maintaining redox balance and supporting glycolysis [30], while glutamate serves as a crucial intermediate in glutamine metabolism, a preferred fuel source for cancer cells [27]. Lastly, pyruvate was included for its essential function in bridging glycolysis and oxidative phosphorylation by feeding into the TCA cycle [14].

### 4.1. Citrate

The relationship between citrate and cancer is multifaceted, with some evidence suggesting that it can act as both a tumor suppressor [56,57,58] and a facilitator of oncogenic processes, depending on the context [25,59]. Our result showed that citrate concentrations were significantly higher in *MEMO1* knockouts compared to *MEMO1*-KD cells across all tested iron conditions and compared to high-*MEMO1* cells at low and basal iron levels. This consistent elevation in citrate levels in *MEMO1*-KO cells suggests that the absence of MEMO1 may disrupt normal metabolic regulation, leading to increased citrate accumulation. The elevated citrate levels in *MEMO1*-KO cells may indicate a metabolic adaptation that favors oxidative phosphorylation and lipid biosynthesis, both of which are essential for tumor growth and survival [60]. This aligns with the concept of citrate as an oncometabolite, where its accumulation can promote de novo lipogenesis, a hallmark of cancer cell metabolism [61]. However, this perspective is complicated by the fact that citrate also acts as an allosteric inhibitor of phosphofructokinase 1 (PFK1) and an activator of fructose-1,6-bisphosphatase (FBP), which inhibits hypoxia-inducible factor 1α (HIF-1α), thereby reducing glucose uptake and glycolytic flux. This dual role has been proposed as a tumor-suppressive mechanism in breast cancer cells [62].

Interestingly, the most obvious inverse correlation between *MEMO1* expression levels and citrate concentration was observed under basal iron conditions, whereas this relationship appears to be more complex both under elevated and depleted iron conditions, suggesting additional interactions between MEMO1 and other regulatory factors when iron homeostasis is disrupted.

### 4.2. Fumarate

Fumarate modulates mitochondrial metabolism through the succination of key proteins involved in iron-sulfur cluster biogenesis, leading to impaired respiratory chain complex I assembly [63]. Fumarate also inhibits ACO2 activity in a dose-dependent manner through succination, thus interfering with iron coordination, which consequently affects the TCA cycle [64].

Similar to citrate, our findings suggest an inverse relationship between *MEMO1* expression level and fumarate concentration; however, unlike citrate, this trend was most pronounced under the low iron conditions, and, to a smaller extent, at the elevated iron level. Under basal conditions, no differences in fumarate levels were observed among the cell lines, indicating that MEMO1 may primarily influence fumarate metabolism under altered iron conditions, which may be attributed to fumarate’s role in redox imbalance. Fumarate can stabilize HIF-1α and succinate glutathione, leading to increased reactive oxygen species levels [64,65]. Given MEMO1 modulatory role in iron homeostasis, *MEMO1*-deficient cells under conditions of iron deregulation may experience redox balance disruption through increased fumarate content.

### 4.3. Malate

Our analysis revealed that malate levels were elevated in *MEMO1* knockout cells only under iron-limiting conditions. This altered malate level under iron depletion may suggest the disruption of MEMO1-dependent iron homeostasis in *MEMO1*-KO cells which could affect the malate-aspartate shuttle. This shuttle is crucial for transferring reducing equivalents between the cytosol and mitochondria, supporting ATP production and maintaining metabolic equilibrium, thus sustaining oxidative metabolism [66]. Supporting this idea, our data indicate a GOF-GI between *MEMO1* and *MDH1*, which encodes malate dehydrogenase 1, a key enzyme in the shuttle that catalyzes the reduction of NAD⁺ to NADH [14]. This finding implies that MEMO1 loss may impair MDH2 activity, potentially reducing the shuttle efficiency. Additionally, the accumulation of malate under iron deficiency may serve as an adaptive response to oxidative stress, as malate can be converted to pyruvate by malic enzyme, generating NADPH to counteract reactive oxygen species [67].

### 4.4. α-Ketoglutarate

Our results for α-KG concentrations exhibited a trend similar to that observed for citrate levels: α-KG was significantly increased in *MEMO1*-KO cells, especially under basal iron conditions, while comparable levels of α-KG were found in both *MEMO1* knockdown and high-*MEMO1* expressing cells under all tested conditions.

The similarity in citrate and α-KG levels trends might be related to their functional connection, as the levels of both metabolites can be influenced by glycolysis and both of them play key roles in regulating the balance between glycolysis and OXPHOS [22,68,69].

Additionally, citrate and α-KG are metabolically interconnected, particularly in tumor cells with compromised mitochondrial function. In such cases, reductive carboxylation of glutamine-derived α-KG serves as an alternative pathway for citrate production in an NADPH-dependent manner, supporting biosynthetic and bioenergetic demands [24].

The consistent rise in α-KG in *MEMO1*-KO cells may indicate that MEMO1 is associated with α-KG homeostasis, potentially independent of iron fluctuations. This regulation could occur through modulating glutamine anaplerosis or α-KG oxidation, contributing to metabolic stability [27,30]. It may also reflect a potential tumor-suppressive function, as α-KG can activate α-KG-dependent dioxygenases, promoting chromatin remodeling and influencing gene expression patterns that favor differentiation [70].

### 4.5. Glutamate

Glutamate levels in the studied cells followed the pattern observed for malate, with a significant increase detected in *MEMO1*-KO cells under iron depletion. In the context of MEMO1’s role in iron regulation, this rise in glutamate may represent an adaptive response to oxidative stress induced by iron deficiency. Elevated glutamate can be converted to α-KG, supporting biosynthetic pathways and sustaining energy production.

Reduced iron availability may also affect electron transport chain proteins, prompting the cell to rely more on the malate–aspartate shuttle to maintain energy production and redox homeostasis. The increased glutamate levels likely support the shuttle’s function by ensuring a sufficient substrate supply for GOT2-mediated transamination.

### 4.6. Pyruvate and Succinate

All three cell lines with varying *MEMO1* expression exhibited unchanged levels of pyruvate and succinate acid across all three iron conditions. The consistent levels of these metabolites, regardless of iron availability or *MEMO1* expression indicate strong metabolic stability related to these two key intermediates.

Since the regulation of pyruvate metabolism is critical for sustaining glycolytic flux and overall metabolic homeostasis [14,71], the stable pyruvate levels may reflect the flexibility and redundancy of major metabolic pathways. Indeed, cells are likely to employ alternative anaplerotic mechanisms to replenish the TCA intermediates without significantly altering the pyruvate pool. Alternatively, the observed stability of pyruvate levels may indicate that MEMO1 impact on cellular metabolism occurs downstream of pyruvate or through pathways that do not significantly perturb pyruvate homeostasis.

Similarly, the unchanged levels of succinate may suggest the activation of compensatory mechanisms within the TCA cycle that maintain a stable succinate pool, independent of *MEMO1* expression or iron availability. Succinate, a key intermediate in the TCA cycle, not only plays a metabolic role but also functions as a crucial signaling molecule with diverse implications for tumorigenesis. As an oncometabolite, succinate inhibits α-ketoglutarate-dependent dioxygenases, leading to DNA hypermethylation and altered epigenetic regulation, ultimately driving tumorigenesis [39,72]. It also acts as a ligand for SUCNR1 in cancer cells, activating the AKT/mTOR/HIF-1α signaling axis and further promoting tumor progression [73]. Given its dual role as both a metabolic intermediate and a signaling molecule, maintenance of a stable succinate concentration likely reflects a cellular strategy to buffer metabolic fluctuations and sustain survival under changing conditions.

In conclusion, our results indicate that MEMO1 contributes to the modulation of cancer cell metabolism in response to iron availability. The observed metabolic shifts in the TCA cycle intermediates, particularly citrate, α-KG, malate, fumarate, and glutamate, may highlight potential adaptive mechanisms related to oxidative phosphorylation, redox balance, and glutamine metabolism. Stability of pyruvate and succinate levels indicates tightly regulated compensatory mechanisms. Further research is needed to fully elucidate how MEMO1 orchestrates these metabolic adaptations in light of its iron-binding activity and the impact of these adaptations on tumor progression.

## Figures and Tables

**Table 1:** TCA-cycle related genes exhibiting gain-of-function interactions with *MEMO1*.

Gene	*p*-Value	Distance Low–High	Protein Name	Dataset
*ACLY*	0.0423	2.5934	ATP Citrate Lyase	Marcotte (BRCA) ^1^ [45]
*ACLY*	0.0079	1.4671	ATP Citrate Lyase	Marcotte (All) ^2^ [46]
*ACLY*	0.0228	0.3913	ATP Citrate Lyase	Achilles (All) [47]
*PCK1*	0.0105	1.3177	Phosphoenolpyruvate Carboxykinase 1	Achilles (BRCA)
*PCK2*	0.0229	0.3120	Phosphoenolpyruvate Carboxykinase 2	DRIVE/Ataris (All) [48]
*OGDH*	0.0382	1.0679	Oxoglutarate Dehydrogenase	Marcotte (All)
*OGDH*	0.0360	0.6191	Oxoglutarate Dehydrogenase	Achilles (All)
*OGDH*	0.0020	0.2184	Oxoglutarate Dehydrogenase	CERES (All) [49]
*MDH1*	0.0416	0.7235	Malate Dehydrogenase 1	Achilles (BRCA)
*ACO2*	0.0319	0.6585	Aconitase 2	DRIVE/Ataris (All)
*ACO2*	0.0380	0.3233	Aconitase 2	CERES (All)
*ACO1*	0.0187	0.1600	Aconitase 1	DRIVE/Ataris (All)
*SUCLA2*	0.0423	0.5430	Succinate-CoA Ligase ADP-Forming Beta Subunit	DRIVE/RSA (BRCA)
*SUCLG1*	0.0127	0.3360	Succinate-CoA Ligase Alpha Subunit	DRIVE/RSA (BRCA)
*PDHB*	0.0097	0.4362	Pyruvate Dehydrogenase E1 Subunit Beta	Marcotte (All)
*PDHA2*	0.0410	0.0529	Pyruvate Dehydrogenase E1 Subunit Alpha 2	CERES (All)
*DLAT*	0.0127	0.4250	Dihydrolipoamide Acetyltransferase	DRIVE/RSA (BRCA)
*IDH3B*	0.0486	0.2790	Isocitrate Dehydrogenase 3 Beta	Marcotte (All)
*IDH3B*	0.0161	0.1010	Isocitrate Dehydrogenase 3 Beta	DRIVE/Ataris (All)
*IDH3A*	0.0421	0.2750	Isocitrate Dehydrogenase 3 Alpha	DRIVE/Ataris (All)
*IDH1*	0.0421	0.2380	Isocitrate Dehydrogenase 1	DRIVE/RSA (All)
*SDHB*	0.0353	0.1842	Succinate Dehydrogenase Iron-Sulfur Subunit	CERES (All)
*SDHC*	0.0110	0.1763	Succinate Dehydrogenase Complex Subunit C	CERES (All)
*DLD*	0.0435	0.1330	Dihydrolipoamide Dehydrogenase	Ataris (All)
*DLD*	0.0353	0.1029	Dihydrolipoamide Dehydrogenase	CERES (All)

^1^ BRCA—breast cancer; ^2^ All—all cancers.

## Data Availability

LC-MS/MS raw data have been deposited in the MetaboLights repository under the accession number MTBLS12348.

## Abbreviations

The following abbreviations are used in this manuscript:

GOF	Gain-of-Function
GOF-GIs	Gain-of-Function Genetic Interactions
*MEMO1*-KD	*MEMO1*-knockdown
*MEMO1*-KO	*MEMO1*-knockout
DmPA	Dimethylaminophenacyl
TCA cycle	Tricarboxylic Acid cycle
OXPHOS	Oxidative Phosphorylation
α-KG	α-Ketoglutarate
LC-MS/MS	Liquid Chromatography–Tandem Mass Spectrometry
HPLC-MS/MS	High-Performance Liquid Chromatography–Tandem Mass Spectrometry
HILIC-MS/MS	Hydrophilic Interaction Liquid Chromatography–Tandem Mass Spectrometry
^12^C_2_-DmPA-Br	^12^C_2_-Dimethylaminophenacyl Bromide
^13^C_2_-DmPA-Br	^13^C_2_-Dimethylaminophenacyl Bromide
MRM	Multiple Reaction Monitoring
ESI	Electrospray Ionization
ISV	Ion Spray Voltage
Q-LTI	Quadrupole-Linear Ion Trap
GSEA	Gene Set Enrichment Analysis
